# Self-care barriers and facilitators in older adults with T1D during a time of sudden isolation

**DOI:** 10.1038/s41598-023-33746-3

**Published:** 2023-04-29

**Authors:** Medha Munshi, Christine Slyne, Katie Weinger, Sarah Sy, Kayla Sifre, Amy Michals, Dai’Quann Davis, Rachel Dewar, Astrid Atakov-Castillo, Saira Haque, M. Stirling Cummings, Stephen L. Brown, Elena Toschi

**Affiliations:** 1 Joslin Diabetes Center, One Joslin Place, Boston, MA 02215 USA; 2 Beth Israel Deaconess Medical Center, Boston, MA USA; 3 Harvard Medical School, Boston, MA USA; 4 RTI International, Research Triangle Park, NC USA

**Keywords:** Endocrine system and metabolic diseases, Psychology

## Abstract

Older adults with type 1 diabetes (T1D) have unique challenges and needs. In this mixed-methods study, we explored the impact of isolation during a pandemic on diabetes management and overall quality of life in this population. Older adults (age ≥ 65 years) with T1D receiving care at a tertiary care diabetes center participated in semi-structured interviews during COVID-19 pandemic isolation between June and August 2020. A multi-disciplinary team coded transcripts and conducted thematic analysis. Thirty-four older adults (age 71 ± 5 years, 97% non-Hispanic white, diabetes duration of 38 ± 7 years, A1C of 7.4 ± 0.9% (57.3 ± 10.1 mmol/mol) were recruited. Three themes related to diabetes self-care emerged regarding impact of isolation on: (1) diabetes management and self-care behaviors (how isolation prompted changes in physical activity and dietary habits); (2) emotional stress and anxiety (related to isolation and lack of support system, economic concerns); and (3) concerns regarding the COVID-19 pandemic (impact on timely medical care and access to information). Our findings identify modifiable barriers and challenges faced by older adults with T1D during isolation. As this population has a higher risk of decline in physical and psychosocial support even during non-pandemic times, clinicians will benefit from understanding these issues to improve care of this population.

## Introduction

Older adults with type 1 diabetes (T1D) are a unique population who have managed a complex disease, often for many decades^1,2^. This cohort followed rigorous diabetes self-care regimens, such as disciplined dietary intake, exercise programs, and complex insulin treatments for many decades^3^. However, as these individuals age, they may acquire some age-related decline in physical and cognitive status as well as other medical comorbidities^4–9^. Further, older adults with T1D face additional challenges in their clinical, functional, and psychosocial environment such as acute illnesses, hospitalizations, caregiver burdens, financial difficulties, and changing support systems^10^. Very little information about how older adults with T1D cope with, and manage their diabetes and self-care activities during difficult periods in their later life is available.

Recently, the lockdown due to the COVID-19 pandemic has affected everyone. During this time, over 160 older adults with T1D were participating in a study at our institution, which was paused. Thus, a unique opportunity arose to understand how older adults with significant medical and social needs manage their T1D when their support system network is disrupted. We used qualitative approaches and methods to understand the impact of isolation and a fractured social network on their ability to care for diabetes, along with quality of life parameters.

## Methods

We performed semi-structured interviews with a convenience sample of 34 older adults of an ongoing study titled “Technological Advances in Glucose Management in Older Adults (TANGO)” assessing the use of CGM in older adults with T1D (Clinicaltrials.gov NCT03078491). Data were collected between May 2020 and August 2020. Eligibility criteria for this interview study included participation in the TANGO study and willingness and capability to participate in this interview study. Everyone in the study provided verbal informed consent. The Joslin Diabetes Center Institutional Review Board approved the study protocol.

The interviews included four broad questions with probes and 23 survey questions. Please see supplement 1 for the interview guide, which was developed with guidance from the psychologist with extensive experience in qualitative research. Each interview was conducted by a research assistant via phone, digitally audio-recorded and lasted 30–60 min. The interviews were later transcribed, coded using NVivo12, and analyzed using qualitative content analysis by categorizing key words and phrases to identify themes. To achieve investigator triangulation, a multidisciplinary team of eight individuals including geriatricians, endocrinologists, a psychologist, a health informaticist, and research assistants individually coded the interviews to identify the themes via bi-weekly meetings over a 6-month period. In the event of disagreement when coding, a majority of the group needed to agree before moving forward.

All names of clinical providers, medical supply companies, medications and brand names of device companies were omitted in the transcript and replaced by either initials or generic names. For each quotation, age; gender; and duration of T1D are provided. Descriptive statistics for demographic and clinical data are reported as number (n) and percentage (%) of the cohort for categorical variables. For continuous variables, data are reported as mean ± standard deviation.

### Ethical approval

The Joslin Diabetes Center’s Institutional Review Board approved the study protocol (CHS # 2016–29). This study was performed in accordance with the Declaration of Helsinki.

## Results

Thirty-four older adults (mean age 71 ± 5 years, T1D duration 38 ± 17 years, mean A1C 7.4 ± 0.9% [57.3 ± 10.1 mmol/mol], 53% women) were interviewed. Table 1 shows the characteristics of this group. Three themes related to impact of isolation on diabetes self-care, emotional stress and anxiety, and COVID-19 related concerns emerged from a thematic analysis. Table 2 provides themes and subthemes of the impact of isolation during the pandemic on self-management and psychosocial aspects.

**Table 1:** Demographics and clinical characteristics of study population.

Demographics	Total number = 34 N (%)
Age (years)	71 ± 5
Female	18 (53)
Ethnicity (White)	33 (97)
Currently working	8 (24)
Some college or higher-level education	32 (94)
History of depression	10 (30)
Number with Cognitive dysfunction (MoCA < 26)	19 (56)
Survey/Interview	
Living with person involved in their diabetes care	19 (38)
Worried about their finances	10 (29)
Had to stop volunteering	10 (29)
Lost any services due to COVID-19	
YMCA, gym, religious services	3 (9)
Most helpful source for gathering information (free response but was categorized for table)	
Media (TV, News, Radio, Newspaper)	19 (56)
Health Officials	7 (21)
None	3 (9)
Felt they needed more information/couldn’t find what they were looking for	8 (24)
Level of confidence in the information you received	
Somewhat confident	19 (56)
Extremely confident	15 (44)
Level of physical activity has changed	26 (76)
Following usual dietary patterns	30 (88)
Connecting with loved ones	
More than usual	13 (38)
Less than usual	6 (18)
The same as usual	14 (41)

**Table 2:** Themes and subthemes of impact of isolation during pandemic on self-management and psychosocial aspects.

Theme	Content area	Positive perspective	Negative perspective
Diabetes self-care	Exercise	Better glucose control due to more time to do more exercise (8)*	Less exercise due to lack of access to venue of choice (9)
	Nutrition	Better eating due to controlled eating environment ( eating at home) and more time (7) impact of good routine and standard practices to fall back on (8)	More eating due to being at home, boredom (4)
	Stress of pandemic/lockdown affecting self-care	No stress about diabetes self-care due to isolation (8)	More stress in managing diabetes with inability to go out(3)
Emotional stress and anxiety	Isolation and lack of usual support system	Continued family support and more time with them (3), more check-ins from family (2), finding ways to keep connected with family and friends outdoors(4) via zoom or phones (9)	Separation of family and friends and lack of physical contact (9), loss of comfortable routine (4)
	Overall anxiety and concern about societal unrest	Keeping busy, being creative to avoid stress (4)	Anxiety about society and state of unrest (6) Burnout from COVID-19- related news (4)
	Economic insecurity	Financial security with investments and other income (5) and unemployment (2)	Concern about savings and stock-market (4)
Concerns regarding COVID-19 pandemic	Fear and anxiety regarding impact of COVID-19	Taking infection mitigating practices in stride without unusual anxiety or fear (14)	Vulnerability to infection and poor outcomes due to diabetes(6), worries about isolation protocols (3)
	Impact of COVID-19 on other medical care	Feeling secure with getting medical care of other conditions as needed (5)	Delay in care regarding other medical needs, surgeries, dental care, eye care, other appointments (11)
	Access to information about health and related topics	Feel comfortable with which source of information to trust (6), Felt comfortable with information from TV (2), government sources local and CDC (5)	Fluid nature of COVID-19-related guidance and information (8) Lack of trust in news source (5) social media (4)

*Number of people in the study who discussed each perspective listed in parentheses.

Theme 1: Impact on diabetes management and self-care behaviors (how isolation prompted more or less physical activity and a change in dietary habits).

Older adults in this study were almost equally divided regarding the positive or negative impact of isolation on their self-care behaviors. Many used the added time at home to improve diet by cooking more healthy meals at home. “Not being able to go out to eat, I was able to better prepare meals for myself. And therefore, I think my diabetes was in better control because of it” (69 year-old woman, T1D 31 years). “One easy thing about the management is, I’m not going to restaurants… Restaurants tend to just really, throw off my numbers. I can never get them right, because of the fat and whatever else they’re adding into sauces” (72 year-old woman, T1D 22 years).

However, some people described the opposite effect of cooking at home. “I am someone who, on average, went out or did take out four or five times a week, and now, I’m actually cooking things, and clearly eating too much of what I cook because I don’t want to waste anything,” (72 year-old man, T1D 33 years).

Regarding physical activity, several people did better than usual, “I’ve been doing a lot more outside walking than I customarily do. I’m trying” (69-year-old woman, T1D 50 years). Others, with more home-based exercise routines, were able to continue without problems. “We live in a community where we can get out and we can walk and exercise a little bit without seeing anybody else. So we actually are doing quite well.” (71 year-old woman, T1D 14 years). However, some found it difficult to change exercise routines, as the gyms and indoor exercise venues were not available. “I was really enjoying exercising at one of the branches of the YMCA, and I lost that, at this point I can’t do that” (73 year-old woman, T1D 26 years).

In addition to diet and exercise, isolation impacted the overall comfort in managing diabetes. “There was a little stress in the beginning, and I did see a few higher glucose numbers back when they first announced that they were going to close things down, and that sort of thing. It was a little nerve-wracking. But that is quickly straightened out” (75 year-old woman, T1D 31 years).

However, a few people in the study remained comfortable in their diabetes management. “Because I’m not running around like I used to, and I feel very relaxed, my husband and I are really bonding together” (72 year-old woman, T1D 22 years). Overall, people who described having structured routines to fall back on reported doing well,

“I made sure the first thing that I needed to do….was to create a routine because I have to manage diabetes medications. I also have to manage Parkinson’s medications and they require different things actually. So, I knew I have to have routine with regular meals and regular exercise. And I have regular contact with my grandchildren and my friends. And so, that gets me through” (70 year-old woman, T1D 13 years).

Theme 2: Emotional stress and anxiety (related to isolation and lack of usual support system, higher risk of COVID-19 due to diabetes, economic concerns, and overall societal unrest).

The impact of isolation was clearly felt in the context of physical separation from family and friends. Several people emphasized comfort with their loved ones checking in on them more frequently. “I would say [my loved ones are checking in] more because, I mean, a lot of my friends call me to see how I’m doing” (73 year-old woman, T1D 26 years). However, many voiced disappointment and distress around not being able to see their loved ones in person, especially their grandchildren, as they are growing up. “I’m seeing visibly less of my grandchildren. I didn’t see them for months, couple of months. Though… we went to their house and sat in the driveway, but there’s less contact, real life contact with my family and friends” (70-year-old woman, T1D 13 years).

In general, people with better family connections and support systems reported less stress and anxiety. One person noted: “I’m lucky that I have my entire family with me, who will just bounce me around and say, ‘…We’ll take care of you. You don’t have to worry about it. You don’t have to do anything’” (65 year-old woman, T1D 29 years).

Many in the cohort adapted their behaviors, and thus reported handling the anxiety related to the COVID-19 pandemic.

“I’m doing fine as far as making adjustments and being emotionally comfortable regarding the COVID-19 virus, it is challenging, but intellectually I understand what’s going on and just realize that certain adjustments need to be made and I’m willing to do them reasonably comfortably” (67 year-old man, T1D 43 years).

Importantly, some people worried about being particularly vulnerable due to having diabetes. “I can have good management but if I got out to see people, my diabetes, it makes me so vulnerable to COVID… It is diabetes that scares me the most about going out” (65 year-old woman, T1D 29 years). This anxiety was overwhelming for some. “Yes. I am [lonely], because with all of the other medical issues that I have, I am very sure I would not survive it. I don’t think my body could fight it” (79 year-old woman, T1D 59 years).

Economic insecurity did not emerge as a common stress area in our cohort, as people in the study reported a lack of concern associated with being retired and having investment income/pension. “Fortunately, my husband was a very intelligent man and he did very well with investing” reported a 79 year-old woman (T1D 59 years). Additionally, some individuals reported unemployment income as helpful. “I am collecting, so that part is good. I am not really financially out of black too much. I am collecting unemployment” (66 year-old woman, T1D 12 years). However, financial market volatility created anxiety for a few in the study. “All my retirement I have in the stock market. I am a little bit nervous about that” (71 year-old man, T1D 37 years).

Pandemic-related and civil unrest-related anxiety was clearly felt, adding to the overall anxiety.

“The world around me has become very challenging [because] of the restrictions placed on me by the COVID-19 virus, then the anxiety created by the social unrest, and then there are some personal issues in my life not related to my health that have just all come together in [a] difficult situation” (69 year-old woman, T1D 50 years).

These issues impacted the overall health of the cohort, “I tend to be anxious anyway, and so during this time I felt I needed to increase my medications for anxiety and depression…” (69 year-old, T1D 31 years).

Theme 3: Concerns regarding the COVID-19 pandemic (related to fear and anxiety regarding impact of COVID-19 on timely medical care and access to reliable trustworthy information).

A number of older adults felt their other medical care was impacted during this time. Uncertainty regarding the timeline of visits and the fluid nature of COVID-19-related guidelines and restrictions were stressful.

“I was supposed to have an appointment with [a provider] and then he stopped all face-to-face appointments. And then when he started up again, it was going to be six months till I could actually see him again. He just moved his whole calendar six months or three months rather than first come first serve. So, that was pretty bad” (68 year-old man, T1D 50 years).

In contrast, some felt their medical needs were met during the pandemic. “I think it’s better. I mean, everybody’s aware of how things fall through the cracks, and so they’re being more vigilant” (76 year-old man, T1D 22 years).

Just like many people in the country during that time, trusting information sources was a concern for some. Many individuals discussed trusting medical professionals as their information source: “Probably from the scientists on TV. Definitely not from the politicians. I only listen to the scientists” (69 year-old woman, T1D 31 years). Another older adult noted she got her information “on TV town halls that they have been having with the doctors and scientists” (72 year-old, T1D 22 years).

A few people, however, did worry about how to analyze the information. “Unfortunately, as we learn more about the virus, the information changes. And while I may feel very confident about it on Monday, by Friday I’m not very confident” (69 year-old woman, T1D 50 years).

Several people complained of burnout from news related to both COVID-19 and social unrest. “I find that it gets overwhelming at times, especially with the news, so I think probably I haven’t watched the news as much, because it will make you feel down when they are talking about so much stuff and there’s got to be a silver lining here” (70 year- old woman, T1D 25 years).

## Discussion

This qualitative study was conducted during the COVID-19 pandemic lockdown to understand the perspective of older adults with T1D when there was a sudden disruption in daily activity, causing isolation and a fractured support system. Although such isolation due to a pandemic is not likely to occur frequently, older adults face similar challenges to their support system in other situations, such as the loss of spousal or caregiver support, isolation due to a decline in cognitive and physical abilities, or anxiety due to new medical diagnoses. Understanding the impact of these challenges on diabetes self-care may help clinicians to develop coping strategies as they care for this unique population of older adults with T1D.

Overall, we found many in the study coped well during these new challenges and maintained a positive attitude. This success and optimism might be due to their inherent capacity to cope with difficult situations, as many have done since being diagnosed with T1D many decades ago. However, people in the study identified several areas that would benefit from additional attention and support. The lessons learned from their perspectives can be applied to other older adults with diabetes.

A majority of the cohort adapted well to COVID-19 restrictions and isolation, and maintained or expanded their exercise and nutrition routines. This ability to adapt seemed to originate from many years of following a routine of disciplined self-care. In fact, many expressed feelings that the controlled environment of being homebound provided better eating habits and more time to do exercise, resulting in improvement in their glucose control. A negative impact on exercise routines was attributed mostly to their inability to access familiar exercise venues, such as the gym or YMCA. Some voiced boredom with their food choices as they were not going out to eat, though they felt they achieved better glucose levels with home-cooked meals. Thus, identifying older individuals without physical activity or dietary routines, and recommending adjustments that fit their current situation is key to successful diabetes self-management during periods of isolation.

Emotional stress in our cohort primarily stemmed from separation from family and friends, and lack of physical contact with others. Isolation due to loss of friends and family members to diseases and death are common as people age. Gradual isolation that follows such circumstances can lead to dysthymia or depression, which ultimately may impact a person’s self-care abilities^11,12^. A routine assessment of the signs of isolation, and adaptation in the support system is important for all older adults, but in particular, those ones with T1D who have a significant added burden of disease management complexity. Referral to mental health professionals and/or community resource specialists to help overcome physical/digital isolation can assist elders in coping with such situations.

Our findings regarding access and information concerns in our cohort were also revealing regarding their psychosocial resilience. A majority in the study were able to take COVID-19 infection mitigating practices in stride and maintain medical care, without extreme anxiety or fear. Some level of anxiety was seen, related to overall health care access, which stemmed from the fluid nature of the guidance regarding when they can get medical attention safely. This concern points towards their life-long attention to caring for themselves in a disciplined manner. However, concern about the well-being of society, and the country in general, weighed heavily in light of social unrest.

The limitations of our study include the homogeneous, white, highly-educated population with T1D at a tertiary care diabetes center. Our cohort was better-educated and had more financial resources than average, which is likely due to the geographical nature of the Joslin Diabetes Center, and the resources available to people who choose to be treated at a tertiary care diabetes center.

Our findings show the overall resilience of older people with T1D in caring for their diabetes, even against adverse circumstances. However, during times of isolation, disruption of self-care routines and loss of caregiver support may lead to increased anxiety. Our results will help clinicians to understand the needs and barriers faced by this population. Identifying procedures to overcome these barriers when isolation arises, such as counseling about changing dietary habits and physical activity routines, as well as how to connect with both health care professionals and mental health resources can mitigate the impact of isolation.

## Supplementary Information

Supplementary Information.

## Data Availability

Data will be made available on https://clinicaltrials.gov when applicable.
