# Supplementary material for: Self-care barriers and facilitators in older adults with T1D during a time of sudden isolation
Source: Sci Rep. 2023 Apr 29;13:7026. doi: 10.1038/s41598-023-33746-3 (PMC10148576; doi:10.1038/s41598-023-33746-3)
Supplement: Supplementary file 1 — Supplementary Information. [file 41598_2023_33746_MOESM1_ESM.pdf]

Screening Letter: _________ Staff Initials: ______
Study ID: _______________ Date:____________

Tango Study

Telephone Covid-19 Interview Script

Hello, my name is __________, and I am calling from the Joslin Diabetes Center specifically with the Tango Study. Is this a good time to discuss a new portion of the study with you?

If NO:
Would you have another time that may be better to discuss the study?

> If YES: (schedule another phone call) Thank you for your time. (End of phone call)
> If NO: Thank you for your time and take care. (End of phone call)

If YES:
The new portion of the study is pertaining to the Covid-19 pandemic. This questionnaire is looking to understand how it has affected you with your quality of life and diabetes management. It will consist of 4 interview questions and some survey questions. We expect it to last around an hour of your time. There will be no monetarily compensation for this visit.

Is this something you would be interested in participating in?

If NO:
Thank you for your time and take care. (End of phone call)

If YES:
Excellent! I will tell you a little bit more about the study. (Continue to the addition to study procedure and read the bullet points)

(NOTE: The addition to the study procedure will be sent to the participant upon completion of the phone call)

At this time are there any questions or comments you have? (NOTE: Take notes of questions and answers provided on the addition to study procedure form)

How would you prefer to be sent the information of the addition to the study?

(NOTE: Turn on recorder and confirm participant consent to record while recording. After completing this, you can begin the interview)

Approved by JDC/CHS

Screening Letter: _________ Staff Initials: ______
Study ID: ________________ Date:____________

Addition to Study Procedure

Participant name: ____
Study ID: ________________________

The interview is expected to last up to an hour. It will consist of four interview questions and 23 survey questions. The conversation between you and I will be recorded and will be used to understand how the Covid-19 pandemic has affected you in regards to your quality of life and diabetes management. The recording will be saved in the Joslin Diabetes Center's encrypted study drives that only study staff have access to. Once the data has been extracted from the interview, the recording will be destroyed.

You do not have to participate in this portion of the study to continue in the TANGO study.

Risks:

- There is minimum risk involved. If any of the questions are upsetting or cause discomfort then you can ask the question to be skipped or chose to stop the interview completely.
- No physical risk involved

Compensation and Benefits:

- No guarantee of benefit, but future research studies and subject may benefit from your participation
- No additional monetary compensation outside of the completed TANGO Study Visits

Authorization and Withdraw:

- Participation is voluntary
- You may withdraw your consent at any time and the interview will end
- If you chose to end the interview, you will not be penalized in your participation of the TANGO study
- We will use any information we have already collected from you before the interview ended

Approved by JDC/CHS

Screening Letter: _________ Staff Initials: ______
Study ID: ________________ Date:____________

VERIFICATION OF EXPLANATION

I hereby certify that I have explained to the above-named participant the purpose of the study, the nature of the study procedures, and such foreseeable risks, potential risks, discomforts, and benefits that may result from their participation in this study. This explanation was made in appropriate language. I have advised the above-named participant to contact their primary care doctor regarding his/her participation in this study, if such contact has not been previously made. I have asked the above-named participant if they have any questions and/or concerns regarding this research study or any of the study's procedures, and I have answered his/her questions to the best of my ability.

I hereby certify that I have explained to the above-named participant the nature and purpose of the use and/or disclosure of his/her medical information, including the possibility that his/her medical information may be obtained by others. This explanation was made in appropriate language. I have asked the above-named participant if they have any questions and/or concerns regarding the use and/or disclosure of his/her medical information for the purpose of this research study, and I have answered his/her questions to the best of my ability.

I hereby certify that I have informed the above-named participant that his/her participation in this research study is completely voluntary. To the best of my knowledge, the decisions made by the above-named participant regarding his/her consent and authorization are accurate reflections of his/her personal choices. To the best of my knowledge, the above-named participant has not been coerced or induced into his/her participation in this research study.

____ __________
Signature of Investigator or Investigator's Representative *Date*

____
Investigator or Investigator's Representative (Print Name)

Approved by
JDC/CHS

Screening Letter: __________ Staff Initials: _______
Study ID: _________________ Date:_____________

Open Ended:

1.) Overall, how are you doing during the Covid-19 pandemic?

   a. What is going well?
   b. What is not going well?
   c. How has your daily routine been impacted by Covid-19?
      i. Did you take any precautionary measures to prevent the infection of Covid-19?
   d. How healthy do you feel overall?
      i. Probe: Have you tested positive? If so, tell us about that experience

NOTE TO STUDY TEAM: If the participant discloses any Covid-19 symptoms such as fever, cough, or shortness of breath, study staff will advise them to contact their primary care provider

→ For interviewer: notes below

____

____

____

____

____

____

____

____

____

____

____

____

____

____

____

Approved by JDC/CHS

Screening Letter: _________ Staff Initials: ______
Study ID: ________________ Date:____________

2.) Overall, how are you managing your diabetes during this Covid-19 pandemic?
   a. What successes have you had, specific to diabetes?
   b. What challenges have you faced, specific to diabetes?
   c. Do you feel you have needed more assistance with your diabetes care during this time? If so, how?

→ For interviewer: notes below

____

____

____

____

____

____

____

____

____

____

____

____

____

____

____

Approved by
JDC/CHS

Screening Letter: _________ Staff Initials: ______
Study ID: ________________ Date:____________

3.) How are you doing using your diabetes technology?

a. Do you feel supported in using your technology at this time?
    i. What has helped?

b. Do you feel confident in getting your supplies?
    i. Have you had any problems getting pump/monitoring supplies/insulin?

→ For interviewer: notes below

____

____

____

____

____

____

____

____

____

____

____

____

____

____

____

Approved by
JDC/CHS

Screening Letter: __________ Staff Initials: _______
Study ID: _________________ Date:_____________

1.) How are you coping emotionally during this time?
   a. Do you feel anxious?
      i. If so, what are your concerns?
      ii. How are you handling it?
      iii. Who do you talk to about your anxiety/fears?
   b. Do you feel sad, lonely, or depressed?
      i. If so, how?
      ii. How are you handling it?
      iii. Who do you talk to about your sadness/loneliness/depression?
      NOTE TO STUDY TEAM: Offer to discuss with study physician if participant needs assistance or advice seeking counseling

2.) Do you have any fears related to your diabetes?
      i. Hypoglycemia
      ii. Hyperglycemia
   b. Are you fearful of contracting Covid-19?
   c. Tell me about your stress and anxiety regarding diabetes during this time.

→ For interviewer: notes below

____

____

____

____

____

____

____

____

____

____

____

____

____

Approved by JDC/CHS

Screening Letter: _________ Staff Initials: ______
Study ID: _______________ Date:____________

Survey Questions:

Living Situation:

1.) Has your living situation changed during this time?
   - Yes
   - No
2.) (If yes): How?
   - Changed residencies by moving in with family/friends
   - Changed residencies by moving to second home/vacation house
   - Friend/family has moved in with me
3.) Are you living with the person who usually is involved in your diabetes care?
   - Yes: Who is this person: __________________
   - No
   - N/A
4.) Where are you currently residing:
   - State: ____________________
   - City: _____________________

Financial/ Job Questions:

5.) Are you worried about your finances?
   - Yes
   - No
6.) (If yes): In what ways are you worried?
   - Food security
   - Mortgage/housing/utilities
   - Retirement fund
   - Stocks
   - Other: ____________________
7.) Did your work situation change due to this pandemic?
   - Yes: If So, how:____
   - No
   - N/A
8.) If you volunteered before the pandemic, have you had to stop volunteering?
   - Yes
   - No
   - N/A
9.) Have you lost any services due to the pandemic?
   - Meals on wheels
   - VNA/PT
   - YMCA
   - N/A
   - Other: ___________

Approved by JDC/CHS

10.) Have you had any financial difficulties obtaining your diabetes supplies?
- ○ Yes: If So, how:____
- ○ No

Information gathering questions:

11.) Where do you get your information about the pandemic?
- ○ Internet: (If yes, please specify):
  - ○ CNN, Google, ABC, Etc.
  - ○ Social Media: Twitter, Facebook, Instagram, YouTube
- ○ Radio
- ○ TV (Debriefings, news, etc.)
- ○ Healthcare providers
- ○ Family and friends

12.) What have you found the most helpful in gathering information:

____
____

13.) What other information did you need that you couldn't find?

____
____

14.) Please indicate your greatest frustrations in trying to seek out information regarding Covid-19?
- ○ Difficulty finding information that is specific to my needs (ie: risk of older adults with T1D or other comorbidities)
- ○ Filtering out accurate information
- ○ Overwhelmed by the abundance of information
- ○ Other: (please specify: ______________________________)

15.) How confident are you in the information you received?
- ○ Not confident
- ○ Somewhat confident
- ○ Extremely confident

Approved by
JDC/CHS

Screening Letter: __________ Staff Initials: _______
Study ID: _________________ Date:_____________

Self-Care questions:

16.) Has your level of physical activity changed during this pandemic?
   - Not at all
   - Somewhat
   - Significantly

17.) Are you able to follow your usual dietary patterns?
   - Yes
   - No

18.) Are you snacking more than usual?
   - Yes
   - No

19.) Are you drinking more alcohol more than usual?
   - Yes
   - No

20.) How are you engaging with your social networks (ie: friends and family) during this time?
   - Video calls
   - Phone calls
   - Writing letters
   - Not really staying connected

21.) What have you found to be the easiest/best ways to keep in touch?

____

____

22.) Are you connecting with loved ones more or less than usual?
   - More
   - Less
   - The same

Approved by JDC/CHS

Screening Letter: __________ Staff Initials: _______
Study ID: _________________ Date:_____________

Overall Medical Care:

23.) Have you had contact with your primary care provider?
- o Yes
- o No (did not need to)
- o No (did need to and was unable)
  - o What was the barrier?
    ____

24.) ____ Do you feel confident that your medical needs are being met during this pandemic?
- o Yes
- o No, please elaborate:
  ____
  ____

Approved by JDC/CHS

Screening Letter: _________ Staff Initials: ______

Study ID: _______________ Date:____________

Is there anything else that we did not ask about that has been a problem for you during this pandemic?

→ For interviewer: notes below

____

____

____

____

____

____

____

____

____

____

____

____

____

____

____

**AT THE END OF THE INTERVIEW: **

Thank you for participating in this interview. If you have any worries about contracting COVID-19 or have any other health concerns, please do not hesitate to reach out to your primary care provider for guidance. A reminder that the symptoms of Covid-19 may include fever (>100.4) or feverish, cough, sore throat, shortness of breath, unusual fatigue, chills, body aches, vomiting, diarrhea, nausea, abdominal pain , and/or loss of smell or taste.

Approved by JDC/CHS
